# Supplementary material for: Flavonoid Composition and Pharmacological Properties of Elaeis guineensis Jacq. Leaf Extracts: A Systematic Review
Source: Pharmaceuticals (Basel). 2021 Sep 24;14(10):961. doi: 10.3390/ph14100961 (PMC8549011; doi:10.3390/ph14100961)
Supplement: Supplementary file 1 [file pharmaceuticals-14-00961-s001.zip › pharmaceuticals-1373177-supplementary.pdf]

Flavonoid Composition and Pharmacological Properties of Elaeis guineensis Jacq. Leaf Extracts: A Systematic Review

Supplementary Material:

Table S1
Table S2

Table S1. In vitro studies assessing the effects of the extracts and selected flavonoids from the leaves of *E. guineensis*.

References	Comparators Extract/Residue/ Compound/Positive Control	Pharmacological Assay	Outcomes	Bioactive Properties
[1]	OPLE:<br>-Methanolic extract (MeOH)<br>-Insoluble fraction (INSOL)<br>-Hexane fraction (HEX)<br>-Ethyl acetate fraction (EA)<br>-Water fraction (WATER)<br><br>Positive control:<br>-BHT	DPPH free radical scavenging activity assay	INSOL: IC50 = 6.87 ± 0.11μg/ml.<br>BHT: IC50 = 8.33 ± 0.07 μg/mL<br>MeOH: IC50 = 17.73 ± 0.04 μg/mL<br>EA: IC50 = 29.05 ± 0.22 μg/mL<br>WATER: IC50 = 41.22 ± 0.36 μg/mL<br>HEX: IC50 = 53.12 ± 0.18 μg/mL	Antioxidant activity
		LPO inhibition assay	INSOL: IC50 = 101.45 ± 0.82 μg/ml.<br>BHT: IC50 = 3.91 ± 0.21 μg/mL<br>MeOH: IC50 = 93.20 ± 0.39 μg/mL<br>EA: IC50 = 43.24 ± 0.45 μg/mL<br>WATER: IC50 = 650.01 ± 1.29 μg/mL<br>HEX: IC50 = 100.34 ± 0.10 μg/mL	
		Beta-carotene bleaching assay	INSOL: IC50 = 53.93 ± 0.49 μg/ml.<br>BHT: IC50 = 1.78 ± 0.33 μg/mL<br>MeOH: IC50 = 72.38 ± 0.55 μg/mL<br>EA: IC50 = 47.86 ± 0.02 μg/mL<br>WATER: IC50 = 152.96 ± 1.26 μg/mL<br>HEX: IC50 = 114.72 ± 0.82 μg/mL	
[2]	OPLE:<br>-Aqueous methanolic extract<br><br>Positive control:	DPPH free radical scavenging activity assay	At 1mg/mL:<br><br>OPLE:<br>Percentage inhibition: 50.14 ± 1.711 %<br>IC50 = 814 μg/mL	Antioxidant activity

	-Vitamin E<br>-Vitamin C<br>- BHT<br>-Allopurinol		Vitamin E:<br>Percentage inhibition: 41.00 ± 0.020 %<br><br>BHT:<br>Percentage inhibition: 66.00 ± 0.010 %	
		Xanthine oxidase Inhibitory (XOI) activity	At 100 µg/mL:<br><br>OPLE:<br>Percentage inhibition: 66.41 ± 0.4 %<br>IC50 = 37.48 µg/mL<br><br>Allopurinol:<br>Percentage inhibition: 95.21 ± 0.3%	
		Nitric oxide scavenging (NOS) activity	At 1000 µg/ml:<br><br>OPLE:<br>Percentage of inhibition: 73.07%<br>IC50 = 534.04 µg/mL<br><br>vitamin C:<br>Percentage of inhibition: 91.62%<br>IC50 = 169.22 µg/mL	
		Hydrogen peroxide scavenging activity (HPSA)	OPLE: IC50 = 1052.02 µg/ml.<br>Vitamin C: IC50= 924.26 µg/ml.	
[3]	Extracts prepared from dried OPL:<br>-soluble free phenolic (SFP)<br>-insoluble bound phenolic (ISBP)<br>-esterified phenolic (EFP)<br>-total phenolic extract (TPE)<br><br>Extracts prepared from fresh OPL:<br>-soluble free phenolic (SFP)	DPPH free radical scavenging activity assay	Percentage inhibition:<br><br>Extracts prepared from dried OPL: 56-93%<br>Extracts prepared from fresh OPL: 65-88%<br><br>Except for the ISBP extract prepared from dried OPL, the remaining OPLE prepared from dried OPL exhibited higher free radical scavenging activity	Antioxidant activity

	-insoluble bound phenolic (ISBP)<br>-esterified phenolic (EFP)<br>-total phenolic extract (TPE)		compared to the OPLE prepared from fresh leaves	
[4]	OPLE:<br><br>OPAL M1:<br>OPL were extracted with ethanol; refluxed at 78oC for 2hours<br><br>OPAL M2:<br>OPL were extracted with hexane followed by ethanol at room temperature<br><br>OPAL M3:<br>OPL were extracted with hexane followed by ethanol acidified with 6M HCL at room temperature<br><br>OPAL M4:<br>OPL were extracted with deionised water; refluxed at 100oC for 2hours<br><br>Positive control:<br>-Trolox<br>- BHT	DPPH free radical scavenging activity assay	Percentage inhibition and IC50 values at 0.25 mg/mL:<br><br>Trolox: 95.78%<br>IC50 = 0.127 ± 0.000 mg/mL<br><br>BHT: 78.63%<br>IC50 = 0.134 ± 0.000 mg/mL<br><br>OPAL M1: 36.05%<br>IC50 = 0.416 ± 0.004 mg/mL<br><br>OPAL M2: 25.32%<br>IC50 = 0.651 ± 0.005 mg/mL<br><br>OPAL M3: 18.07%<br>IC50 = 1.330 ± 0.010 mg/mL<br><br>OPAL M1: 15.47%<br>IC50 = 1.306 ± 0.001 mg/mL	
[5]	OPLE:<br>-Hexane extract<br>-Ethyl acetate extract<br>-Ethyl acetate-methanol extract<br>-Absolute methanol extract<br>-Aqueous methanol extract	3-(4,5-Dimethylthiazol-2-yl)-2,5-Diphenyl Tetrazolium Bromide (MTT) assay	No toxicity effects on 3T3 mouse fibroblasts with all cell viability percentage reached above 70%.<br><br>Absolute methanolic, aqueous methanolic, and ethyl acetate-methanolic of OPL extracts: At 25	Cytotoxicity effect

Flavonoid enriched fraction<br><br>Flavonoid C-glycoside enriched fraction<br><br>Flavonoid-C-glycosides:<br>-Orientin<br>-Isoorientin<br>-Vitexin<br>-Isovitexin<br><br>Negative Control:<br>Without treatment<br><br>Positive control:<br>Allantoin		µg/ml, more than 100% of cell viability. There is a sign of decrease in percentage cell viability as the concentration increases.	
2.	Cell proliferation assay	Good proliferative activity (>70%) for all samples at all tested concentrations (1.563 to 12.5 µg/mL)<br><br>Hexane, ethyl acetate, ethyl acetate-methanol and methanol extracts: almost reached 100% cell proliferation rate at all tested concentrations (1.563 to 12.5 µg/mL)<br><br>Aqueous methanol extract: >100% cell proliferation rate at 1.563 µg/mL<br><br>Flavonoid enriched fractions: >120% cell proliferation rate at 3.125 µg/mL<br><br>Flavonoid C-glycoside enriched fractions: >150% cell proliferation rate at 6.25 µg/mL<br><br>Flavonoid C-glycosides: almost 100% cell proliferation rate at 3.125 µg/mL<br><br>Proliferation rate decreased as the concentrations of the samples increased.	Wound healing activity

		All samples did not affect cellular activity of 3T3 fibroblast cells at tested concentrations
	Scratch assay	Enhanced migration and proliferation of 3T3 cells by all the samples after 24 hours<br><br>Migration and proliferation of 3T3 cells by samples after 48h:<br><br>Negative control:<br>40.35%<br><br>Allantoin:<br>94.96% at 1.563 μg/mL<br><br>Absolute methanol extract:<br>95.27% at 3.125 μg/mL<br><br>Aqueous methanol extract:<br>92.56% at 3.125 μg/mL<br><br>Flavonoid enriched fraction:<br>92.56-94.17% at 3.125 μg/mL<br><br>Flavonoid C-glycoside enriched fraction<br>89.54-90.20% at 3.125 μg/mL<br><br>Orientin:<br>98.16% at 3.125 μg/mL<br><br>Isoorientin:<br>87.40-88.89% at 1.563 μg/mL<br><br>Vitexin:<br>87.40-88.89% at 1.563 μg/mL

			Isovitexin<br>87.40-88.89% at 1.563 µg/mL<br><br>3T3 cells migrated better at lower concentration than higher concentration for all samples.	
[6]	Aqueous methanolic plant extract:<br><br>-Green tea (positive control)<br>-Betel Leaves<br>-Cashew<br>-Japanese mints<br>-Semambu<br>-OPL<br>-Sweet potato<br>-Chili<br>-Papaya<br>-Roselle<br>-Maman<br>-Noni<br>-Lemongrass	Inhibition of copper-mediated oxidation of LDL<br>(Conjugated diene formation)	Betel leaves:<br>lag time = 106.9 ± 5.8 mins<br>percentage inhibition: 94%<br><br>Green tea:<br>lag time = 97.0 ± 2.2 mins<br>percentage inhibition: 76%<br><br>Cashew:<br>lag time = 89.9 ± 3.3 mins<br>percentage inhibition: 63%<br><br>Japanese mints:<br>lag time = 84.0 ± 2.0 mins<br>percentage inhibition: 52%<br><br>Semambu:<br>lag time = 82.5 ± 1.4 mins<br>percentage inhibition: 50%<br><br>OPL:<br>lag time = 77.9 ± 0.7 mins<br>percentage inhibition: 41%<br><br>Sweet potato:	Antioxidant activity

		lag time = 69.0 ± 1.8 mins<br>percentage inhibition: 25%<br><br>Chilli:<br>lag time = 67.7 ± 1.8 mins<br>percentage inhibition: 23%<br><br>Papaya:<br>lag time = 59.8 ± 0.8 mins<br>percentage inhibition: 8%<br><br>Roselle:<br>lag time = 59.2 ± 1.3 mins<br>percentage inhibition: 7%<br><br>Maman:<br>lag time = 58.3 ± 0.9 mins<br>percentage inhibition: 6%<br><br>Noni:<br>lag time = 54.8 ± 1.3 mins<br>percentage inhibition: -1%<br><br>Lemongrass:<br>lag time = 54.8 ± 1.7 mins<br>percentage inhibition: -1%
	Inhibition of copper-mediated oxidation of LDL [thiobarbituric acid reactive substance (TBARS) assay]	Betel leaves:<br>percentage inhibition (after 1hour): 90%<br>percentage inhibition (after 2hours): 63%<br><br>Green tea:<br>percentage inhibition (after 1hour): 88%<br>percentage inhibition (after 2hours): 40%

Cashew:
percentage inhibition (after 1hour):
93%
percentage inhibition (after 2hours):
60%

Japanese mints:
percentage inhibition (after 1hour):
77%
percentage inhibition (after 2hours):
16%

Semambu:
percentage inhibition (after 1hour):
76%
percentage inhibition (after 2hours):
22%

OPL:
percentage inhibition (after 1hour):
74%
percentage inhibition (after 2hours):
5%

Sweet potato:
percentage inhibition (after 1hour):
46%
percentage inhibition (after 2hours):
3%

Chilli:
percentage inhibition (after 1hour):
47%
percentage inhibition (after 2hours):
11%

			Papaya:<br>percentage inhibition (after 1hour): 20%<br>percentage inhibition (after 2hours): 1%<br><br>Roselle:<br>percentage inhibition (after 1hour): 37%<br>percentage inhibition (after 2hours): 3%<br><br>Maman:<br>percentage inhibition (after 1hour): 5%<br>percentage inhibition (after 2hours): 3%<br><br>Noni:<br>percentage inhibition (after 1hour): 5%<br>percentage inhibition (after 2hours): 5%<br><br>Lemongrass:<br>percentage inhibition (after 1hour): 20%<br>percentage inhibition (after 2hours): 0%	
[7]	OPLE:<br>-Aqueous methanol extract<br>-Absolute methanol extract<br>-Ethyl acetate-methanol extract<br>-Ethyl acetate extract<br><br>Positive control:<br>-Quercetin	Ferric reducing/antioxidant power (FRAP) assay	Aqueous methanol extract: 101.48 ± 16.67 mg AAE/g<br><br>Absolute methanol extract: 71.62 ± 21.05 mg AAE/g<br><br>Ethyl acetate-methanol extract: 94.00 ± 23.94 mg AAE/g	Antioxidant activity

	-Allantoin		Ethyl acetate:<br>16.26 ± 6.65 mg AAE/g	
		DPPH free radical scavenging activity assay	Aqueous methanol extract:<br>IC50 = 3.53 ± 1.30 μg/ml<br><br>Absolute methanol extract:<br>IC50 = 6.54 ± 3.31 μg/ml<br><br>Ethyl acetate-methanol extract:<br>IC50 = 10.74 ± 3.51 μg/ml<br><br>Ethyl acetate extract:<br>IC50 = 67.92 ± 14.16 μg/ml	
		Nitric oxide scavenging (NOS) activity	Aqueous methanol extract:<br>IC50 = 18.77 ± 3.37 μg/mL<br><br>Absolute methanol extract:<br>IC50 = 67.64 ± 21.97 μg/mL<br><br>Ethyl acetate-methanol extract:<br>IC50 = 109.08 ± 29.61 μg/mL<br><br>Ethyl acetate extract:<br>IC50 = 213.34 ± 58.14 μg/mL	
		In vitro wound healing assay	Proliferation (at 1.56 μg/mL):<br><br>Aqueous methanol extract:<br>107.7 ± 13.11%<br>Absolute methanol extract:<br>100.13 ± 1.07%<br>Ethyl acetate-methanol extract:<br>98.41 ± 4.07%<br>Ethyl acetate extract:<br>94.97 ± 2.12%<br><br>Migration (at 1.56 μg/mL):<br>Aqueous methanol extract:	Wound healing activity

			93.34 ± 4.35%<br>Absolute methanol extract:<br>88.56 ± 6.94%<br>Ethyl acetate-methanol extract:<br>85.83 ± 3.25%<br>Ethyl acetate extract:<br>64.34 ± 1.68%	
[8]	-Aqueous methanolic OPLE<br>-Aqueous methanolic extract of green chilli<br>-Aqueous methanolic extract of papaya shoot<br>-Aqueous methanolic extract of lemongrass<br><br>Vehicle control (for FRAP & In vitro LDL oxidation assays)	Ferric reducing/antioxidant power (FRAP) assay	OPL: 203 ± 4 μmol<br>Green chili: 43 ±2 μmol<br>Papaya shoot: 58 ± 1 μmol<br>Lemongrass: 22 ± 1 μmol	Antioxidant activity
		In vitro LDL oxidation	Greater increase in the lag time of extracts of papaya shoots, green chilli and OPL compared to vehicle control<br><br>The longest delay occurred with OPLE (77.9 ± 0.6 min) compared with vehicle control (55.2 ± 0.5 min).	Antioxidant activity
[9]	Positive control:<br>-Green tea extract<br><br>OPLE:<br>Methanolic extract<br>Chloroform extract<br>Acetone extract<br>Hexane extract<br>Petroleum ether extract<br><br>Test compounds:<br>-Luteolin<br>-Kaempferol<br>-Quercetin<br>-Myricetin<br>-Apigenin<br>-Rutin<br>-Catechin	Ferric reducing/antioxidant power (FRAP) assay	Green tea extract:<br>4.8 ± 0.1 mmol FRAP/g dry sample<br><br>Methanolic OPLE:<br>1.1 ± 0.1 mmol FRAP/g dry sample<br><br>Luteolin:<br>8.4 ± 0.8 mmol FRAP/g sample<br><br>Kaempferol:<br>6.5 ± 0.1 mmol FRAP/g sample<br><br>Quercetin:<br>14.7 ±0.6 mmol FRAP/g sample<br><br>Myricetin:<br>7.7 ± 0.3 mmol FRAP/g sample	Antioxidant activity

	-Epicatechin<br><br>Positive control:<br>- α-tocopherol		Apigenin:<br>0.2 ± 0.0 mmol FRAP/g sample	
		β-carotene-linoleic acid bleaching assay	Precentage inhibition:<br><br>α-tocopherol: 94.6 ± 0.4%<br>Luteolin: 76.7 ± 3.1%<br>Kaempferol: 30.1 ± 1.6%<br>Quercetin: 23.1 ± 0.5%<br>Myricetin: 8.2 ± 2.2%<br>Apigenin: 5.1 ± 0.8%<br>Rutin: 3.1 ± 2.1%<br>Catechin: 24.2 ± 5.5%<br>Epicatechin: 26.4 ± 0.8%<br>Methanolic extract: 25.8 ± 4.9%<br>Chloroform extract: 28.2 ± 1.8%<br>Acetone extract: 22.8 ± 3.5%<br>Hexane extract: 19.4 ± 6.2%<br>Petroleum ether extract: 16.8 ± 4.4%	
[10]	OPLE:<br>-Ethanolic extract<br>Positive control:<br>-Vitamin C<br>- BHT	Hydrogen peroxide scavenging activity (HPSA)	OPLE: EC50 = 72 μg/ml.<br>BHT: EC50 = 32 μg/ml.<br>Vitamin C: EC50 = 40 μg/ml	Antioxidant activity
		DPPH free radical scavenging activity assay	OPLE: EC50 = 35 μg/ml.<br>BHT: EC50 = 11 μg/ml.<br>Vitamin C: EC50 = 18 μg/ml.	
		LPO inhibition assay	OPLE: EC50 = 140 μg/mL<br>Vitamin C: EC50 = 28 μg/mL.	
[11]	OPLE:<br>-Ethanolic extract<br><br>Positive control:<br>-Ciprofloxacin	Anti-yeast activity:<br><br>Disc diffusion assay<br><br><br>Broth microdilution technique	Zone of inhibition:<br><br>OPLE: 16 mm at 10 mg/mL<br>Ciprofloxacin: 21 mm at 5 μg/mL<br><br>OPLE: MIC value of 6.25 mg/mL	Fungicidal activity

Table S2. In vivo studies assessing the effects of the OPLE, and selected flavonoids identified in the leaves of *E. guineensis*.

Citations	Animal Model	Comparators	Assays/Tests	Outcomes	Bioactive Properties
[12]	Female Sprague-Dawley rats	-Normal control rats (NC)<br><br>-OVX control rats (OVX)<br><br>-OVX rats supplemented with 2% green tea (OVX+GT)<br><br>-OVX rats supplemented with OPL 150 mg/kg body weight (OVX + OPL-150)<br><br>-OVX rats supplemented with OPL 300 mg/kg body weight (OVX + OPL-300)	Ash weight of femur and tibia	NC:<br>Femur: 0.34g<br>Tibia: 0.24g<br><br>OVX:<br>Femur: 0.28g<br>Tibia: 0.22g<br><br>OVX+GT:<br>Femur: 0.35g<br>Tibia: 0.23g<br><br>OVX + OPL-150:<br>Femur: 0.38g<br>Tibia: 0.25<br><br>OVX + OPL-300:<br>Femur: 0.37g<br>Tibia: 0.26<br><br>OPL-150 and OPL-300 with increased femur (+30%) and tibia (+20%) ash weights compared with OVX.	Phyto-estrogenic properties, estrogenic activity, osteogenic activity
			Wet and dry masses of femur and tibia	Femur (wet):<br><br>NC:<br>Mass: 0.78 ± 0.02g<br>Change: 0%<br><br>OVX:<br>Mass: 0.74 ± 0.01g	

Change: -5.1%

OVX+GT:
Mass: 0.78 ± 0.02g
Change: +5.4%
OVX + OPL-150:
Mass: 0.80 ± 0.01g
Change: +8.1%

OVX + OPL-300:
Mass: 0.80 ± 0.02g
Change: +8.1%

Tibia (wet):

NC:
Mass: 0.62 ± 0.02g
Change: 0%

OVX:
Mass: 0.60 ± 0.01g
Change: -3.2%

OVX+GT:
Mass: 0.64 ± 0.01g
Change: +6.7%

OVX + OPL-150:
Mass: 0.67 ± 0.01g
Change: +11.7%

OVX + OPL-300:
Mass: 0.67 ± 0.01g
Change: +11.7%

	OPL-150 and OPL-300 increased femur (+8.1%) and tibia (+11.7%) wet masses compared with OVX.<br><br>Femur (dry):<br><br>NC:<br>Mass: 0.58 ± 0.01g<br>Change: 0%<br><br>OVX:<br>Mass: 0.57 ± 0.01g<br>Change: -1.7%<br><br>OVX+GT:<br>Mass: 0.58 ± 0.01g<br>Change: +1.8%<br><br>OVX + OPL-150:<br>Mass: 0.59 ± 0.02g<br>Change: +5.3%<br><br>OVX + OPL-300:<br>Mass: 0.60 ± 0.01g<br>Change: +3.5%<br><br>Tibia (dry):<br><br>NC:<br>Mass: 0.43 ± 0.01g<br>Change: 0%<br><br>OVX:<br>Mass: 0.42 ± 0.01g<br>Change: -2.3%<br><br>OVX+GT:

		Mass: 0.44 ± 0.01g<br>Change: +4.8%<br><br>OVX + OPL-150:<br>Mass: 0.46 ± 0.01g<br>Change: +7.1%<br><br>OVX + OPL-300:<br>Mass: 0.45 ± 0.01g<br>Change: +9.5%<br><br>OPL-300 with increased femur (+3.5%) and tibia (+9.5%) dry masses compared to OVX.<br><br>OPL-150 with increased femur (+5.3%) and tibia (+7.1%) dry masses compared to OVX.	
	Total calcium content	NC:<br>Femur: 2.80mg<br>Tibia: 2.74mg<br><br>OVX:<br>Femur: 2.78mg<br>Tibia: 2.67mg<br><br>OVX+GT:<br>Femur: 2.78mg<br>Tibia: 2.76mg<br><br>OVX + OPL-150:<br>Femur: 2.86mg<br>Tibia: 2.82mg<br><br>OVX + OPL-300:<br>Femur: 2.86mg<br>Tibia: 2.80mg	

T-ALP level	NC:<br>Level: 258.67 ± 1.57 U/L<br>Change: 0%<br><br>OVX:<br>Level: 154.67 ± 1.45 U/L<br>Change: -40.2%<br><br>OVX+GT:<br>Level: 188.00 ± 3.54 U/L<br>Change: +21.5%<br><br>OVX + OPL-150:<br>Level: 196.33 ± 4.04 U/L<br>Change: +26.9%<br><br>OVX + OPL-300:<br>Level: 272.33 ± 3.80 U/L<br>Change: +76.1%<br><br>Compared to normal control:<br>OPL-150 increased (+26.9%)<br>OPL-300 significantly increased (+76.1%)<br>OVX significantly decreased (-40.2%)<br>OVX+GT increased (+21.5%)
Serum calcium and phosphorus levels	NC:<br>Calcium: 2.91 ± 0.10 mmol/L<br>phosphorus: 3.11 ± 0.05 mmol/L<br><br>OVX:<br>Calcium: 2.83 ± 0.04 mmol/L<br>phosphorus: 2.96 ± 0.02 mmol/L<br><br>OVX+GT:<br>Calcium: 2.92 ± 0.02 mmol/L

				phosphorus: 3.09 ± 0.03 mmol/L<br><br>OVX + OPL-150:<br>Calcium: 2.84 ± 0.09 mmol/L<br>phosphorus: 2.98 ± 0.04 mmol/L<br><br>OVX + OPL-300:<br>Calcium: 2.90 ± 0.04 mmol/L<br>phosphorus: 2.97 ± 0.03 mmol/L<br><br>No significant differences.	
			Hematoxylin & Eosin staining	Markedly decreased trabecular bone density in OVX compared with NC and OVX + OPL-300.	
[13]	Male Wistar Kyoto rats	Normal control rats<br><br>Normal rats given OPLE<br><br>Normal rats given captopril<br><br>(L-NAME) induced NO-deficient rats and vehicle<br><br>(L-NAME) induced NO-deficient rats and OPLE<br><br>(L-NAME) induced NO-deficient rats and captopril<br><br>.	CA1 region of hippocampus	NO deficiency destroyed 53% of viable pyramidal cells.<br><br>OPLE treatment retained 74% of viable neurons in normal rats.<br><br>Captopril treatment: less effective neuroprotection, lower pyramidal cell count compared to OPLE treatment.	Neuro-protective, neurogenesis effects
			CA3 region of hippocampus	NO deficiency destroyed 80% of viable neurons.<br><br>OPLE treatment retained 72% of viable neurons in normal rats.<br><br>Captopril treatment retained 47% of viable neurons in normal rats.	
			DG region of hippocampus	NO deficiency destroyed 68% of viable granule cells.<br><br>OPLE treatment retained 76% of of viable neurons in normal rats.	

				Captopril treatment retained 56% of neurons in normal rats.	
			Superoxide dismutase (SOD) activity	SOD activity is decreased in L-NAME induced NO deficient rats.<br><br>OPLE and captopril significantly attenuated the decrease in the SOD activity in NO deficient rats.	Antioxidant activity
			Catalase (CAT) activity	CAT activity is decreased in L-NAME induced NO deficient rats.<br><br>OPLE increased the catalase activity by 24% close to normal levels in NO-deficient rats.	
			MDA level	MDA levels and viable neuron count are low in L-NAME induced NO deficient rats.<br><br>OPLE and captopril increased the MDA levels and viable neuron count in NO deficient rats.	
			Glutathione peroxidase (GPx)activity	No significant effects by NO deficiency.	
[14]	Male Sprague-Dawley rats	Normal control rats (NC)<br>Normal rats receiving 100 mg/kg OPL (N100-OPL)<br><br>Diabetic control rats (DC)<br>Diabetic rats receiving 50 mg OPL (D50-OPL)	Body weight changes	Body weight in NC rats:<br>Day 1: 290g<br>Day 35: 300g<br><br>Body weight in DC rats:<br>Day 1: 300g<br>Day 35: 220g<br><br>Body weight in D50-OPL:<br>Day 1: 240g<br>Day 35: 250g	Hypoglycaemic activity

	Diabetic rats receiving 100 mg OPL (D100-OPL)<br><br>Diabetic rats receiving 200 mg OPL (D200-OPL)<br><br>Diabetic rats receiving glibenclamide 30 mg/kgb.w (GB)		Body weight in D100-OPL:<br>Day 1: 250g<br>Day 35: 300g<br><br>Body weight in D200-OPL:<br>Day 1: 250g<br>Day 35: 310 g<br><br>Body weight in N100-OPL rats:<br>Day 1: 270g<br>Day 35: 260g<br><br>Body weight in GB rats:<br>Day 1: 300g<br>Day 35: 230g<br><br>Mortality rate:<br><br>D100-OPL & D200-OPL rats: significantly lower by 71% compared to DC rats.
		Blood glucose level	Blood glucose level in NC rats:<br>Day 1: 5 mmol/L<br>Day 35: 5 mmol/L<br><br>Blood glucose level in DC rats:<br>Day 1: 15 mmol/L<br>Day 35: 22 mmol/L<br><br>Blood glucose level in D50-OPL:<br>Day 1: 17 mmol/L<br>Day 35: 15 mmol/L<br><br>Blood glucose level in D100-OPL:<br>Day 1: 18 mmol/L<br>Day 35: 15 mmol/L

		Blood glucose level in D200-OPL:<br>Day 1: 18 mmol/L<br>Day 35: 10 mmol/L<br><br>Blood glucose level in N100-OPL rats:<br>Day 1: 5 mmol/L<br>Day 35: 5 mmol/L<br><br>Blood glucose level in GB rats:<br>Day 1: 18 mmol/L<br>Day 35: 18 mmol/L	
	Thiobarbituric acid reactive substance (TBARS) assay	NC:<br>0.159 ± 0.034 μM/g protein<br>N100-OPL:<br>0.224 ± 0.038 μM/g protein<br>DC:<br>0.405 ± 0.168 μM/g protein<br>GB:<br>0.156 ± 0.020 μM/g protein<br>D50-OPL:<br>0.181 ± 0.060 μM/g protein<br>D100-OPL:<br>0.070 ± 0.004 μM/g protein<br>D200-OPL:<br>0.185 ± 0.049 μM/g protein	Antioxidant activity
	Catalase (CAT) activity	NC:<br>0.006 ± 0.001 k/s/mg protein<br>N100-OPL:<br>0.006 ± 0.001 k/s/mg protein<br>DC:<br>0.002 ± 0.001 k/s/mg protein<br>GB:<br>0.006 ± 0.001 k/s/mg protein<br>D50-OPL:<br>0.004 ± 0.000 k/s/mg protein<br>D100-OPL:	

		0.006 ± 0.000 k/s/mg protein<br>D200-OPL:<br>0.005 ± 0.002 k/s/mg protein	
	Superoxide dismutase (SOD) activity	NC:<br>1.971 ± 0.173 U/g protein<br>N100-OPL:<br>3.580 ± 1.300 U/g protein<br>DC:<br>0.211 ± 0.056 U/g protein<br>GB:<br>2.021 ± 0.537 U/g protein<br>D50-OPL:<br>1.085 ± 0.247 U/g protein<br>D100-OPL:<br>1.764 ± 0.268 U/g protein<br>D200-OPL:<br>2.449 ± 0.456 U/g protein	
	Histopathology of liver and kidney of diabetic rats	Kidney damage:<br>NC:<br>30.0± 4.8%<br>DC:<br>80.2 ± 5.5%<br>D50-OPL:<br>31.7 ± 3.9%<br>D100-OPL:<br>25.4 ± 3.1%<br>D200-OPL:<br>40.1 ± 5.6%<br><br>Liver necrotic and inflamed cells:<br>NC:<br>54.00 ± 2.08%<br>DC:<br>88.00 ± 1.53%<br>D50-OPL:<br>24.40 ± 2.76%<br>D100-OPL:	

	25.20 ± 1.94%<br>D200-OPL:<br>34.73 ± 1.50a%
Liver and kidney markers<br><br>TG: Triglycerides<br><br>Total Protein<br><br>AST: Aspartate amino transferase<br><br>ALT:<br>alanine amino transferase<br><br>Creatinine	For TG:<br>NC:<br>1.55 ± 0.09 mg/dl<br>N100-OPL:<br>1.71 ± 0.08 mg/dl<br>DC:<br>0.59 ± 0.17 mg/dl<br>GB:<br>0.91 ± 0.17 mg/dl<br>D50-OPL:<br>2,02 ± 0.23 mg/dl<br>D100-OPL:<br>1.65 ± 0.49 mg/dl<br>D200-OPL:<br>2.34 ± 0.28 mg/dl<br><br>For Total Protein:<br>NC:<br>75.38 ± 0.59 g/dl<br>N100-OPL:<br>75.43 ± 0.69 g/dl<br>DC:<br>62.50 ± 5.94 g/dl<br>GB:<br>73.00 ± 0.58 g/dl<br>D50-OPL:<br>77.50 ± 0.76 g/dl<br>D100-OPL:<br>74.56 ± 1.49 g/dl<br>D200-OPL:<br>71.88 ± 0.64 g/dl<br><br><br>For AST:

	NC:<br>211.1± 8.5 U/L<br>N100-OPL:<br>220.4 ± 16.8 U/L<br>DC:<br>439.1 ± 27.2 U/L<br>GB:<br>178.0 ± 7.71 U/L<br>D50-OPL:<br>228.4 ± 13.4 U/L<br>D100-OPL:<br>225.0 ± 13.3 U/L<br>D200-OPL:<br>261.6 ± 5.9 U/L<br><br>For ALT:<br>NC:<br>53.56 ± 3.75 U/L<br>N100-OPL:<br>52.01 ± 2.63 U/L<br>DC:<br>129.8 ± 15.1 U/L<br>GB:<br>89.7 ± 12.1 U/L<br>D50-OPL:<br>78.34 ± 9.37 U/L<br>D100-OPL:<br>79.60 ± 7.69 U/L<br>D200-OPL:<br>64.85 ± 1.27 U/L<br><br>Creatinine:<br>NC:<br>67.38 ± 2.05 μmol/L<br>N100-OPL:<br>60.71 ± 2.26 μmol/L<br>DC:

				57.33 ± 5.33 μmol/L<br>GB:<br>52.60 ± 2.29 μmol/L<br>D50-OPL:<br>64.50 ± 1.70 μmol/L<br>D100-OPL:<br>71.11 ± 4.48 μmol/L<br>D200-OPL:<br>63.13 ± 2.02 μmol/L	
[9]	New Zealand white rabbits	Normal diet (N)<br><br>Cholesterol diet (C)<br><br>Cholesterol + oil palm leaf extract 0.2% (OPLE) diet<br><br>Cholesterol + oil palm leaf powder 1% (OPLP) diet	Serum total cholesterol level	Week 0:<br>N:<br>1.0 ± 0.1 mmol/L<br>C:<br>1.2 ± 0.3 mmol/L<br>OPLE:<br>1.2 ± 0.2 mmol/L<br>OPLP:<br>1.0 ± 0.2 mmol/L<br><br>Week 8:<br>N:<br>1.0 ± 0.4 mmol/L<br>C:<br>20.8 ± 4.8 mmol/L<br>OPLE:<br>1.7 ± 1.3 mmol/L<br>OPLP:<br>1.5 ± 0.8 mmol/L<br><br>Week 16:<br>N:<br>0.5 ± 0.1 mmol/L<br>C:<br>7.2 ± 4.9 mmol/L<br>OPLE:<br>10.6 ± 4.9 mmol/L<br>OPLP:	Hypocholesterolemic activity

	9.5 ± 2.6 mmol/L<br><br>>2 mmol/L considered hypercholesterolemic.
HDL-C level	Week 0:<br>N:<br>0.7 ± 0.1 mmol/L<br>C:<br>0.8 ± 0.1 mmol/L<br>OPLE:<br>0.7 ± 0.1 mmol/L<br>OPLP:<br>0.7 ± 0.2 mmol/L<br><br>Week 8:<br>N:<br>0.5 ± 0.0 mmol/L<br>C:<br>2.2 ± 0.6 mmol/L<br>OPLE:<br>1.1 ± 0.6 mmol/L<br>OPLP:<br>1.4 ± 0.7 mmol/L<br><br>Week 16:<br>N:<br>0.3 ± 0.1 mmol/L<br>C:<br>1.1 ± 0.4 mmol/L<br>OPLE:<br>0.9 ± 0.6 mmol/L<br>OPLP:<br>0.9 ± 0.4 mmol/L
Triglyceride level	Week 0:<br>N:<br>1.1 ± 0.4 mmol/L<br>C:

		1.2 ± 0.4 mmol/L<br>OPLE:<br>1.4 ± 0.7 mmol/L<br>OPLP:<br>1.1 ± 0.3 mmol/L<br><br>Week 8:<br>N:<br>2.3 ± 2.2 mmol/L<br>C:<br>2.1 ± 0.9 mmol/L<br>OPLE:<br>1.5 ± 0.4 mmol/L<br>OPLP:<br>1.6 ± 0.7 mmol/L<br><br>Week 16:<br>N:<br>1.2 ± 0.8 mmol/L<br>C:<br>1.4 ± 0.4 mmol/L<br>OPLE:<br>1.1 ± 0.8 mmol/L<br>OPLP:<br>1.0 ± 0.6 mmol/L<br><br>Week 16:<br>No significant differences between groups.	
	Body and organ weight	Week 0:<br>N:<br>2.8 ± 0.5 kg<br>C:<br>3.2 ± 0.3 kg<br>OPLE:<br>2.9 ± 0.4 kg<br>OPLP:	

2.8 ± 0.2 kg<br><br>Week 8:<br>N:<br>2.8 ± 0.2 kg<br>C:<br>3.5 ± 0.3 kg<br>OPLE:<br>3.1 ± 0.4 kg<br>OPLP:<br>3.2 ± 0.3 kg<br><br>Week 16:<br>N:<br>2.8 ± 0.3 kg<br>C:<br>3.4 ± 0.2 kg<br>OPLE:<br>3.1 ± 0.4 kg<br>OPLP:<br>3.2 ± 0.3 kg<br><br>Liver weight/body weight (g/kg)<br><br>Week 16:<br>N:<br>20.2 ± 1.9 g/kg<br>C:<br>33.8 ± 2.5 g/kg<br>OPLE:<br>26.6 ± 3.9 g/kg<br>OPLP:<br>25.2 ± 5.6 g/kg<br><br>Kidney weight/body weight (g/kg)<br><br>Week 16:

		N:<br>2.2 ± 0.4 g/kg<br>C:<br>2.4 ± 0.2 g/kg<br>OPLE:<br>2.4 ± 0.2 g/kg<br>OPLP:<br>2.2 ± 0.2 g/kg<br><br>Heart weight/body weight (g/kg)<br><br>Week 16:<br>N:<br>2.6 ± 0.4 g/kg<br>C:<br>2.2 ± 0.2 g/kg<br>OPLE:<br>2.6 ± 0.4 g/kg<br>OPLP:<br>2.4 ± 0.2 g/kg	
	Catalase (CAT) activity	Week 0:<br>N:<br>0.2 ± 0.0 k/mg Hb<br>C:<br>0.2 ± 0.1 k/mg Hb<br>OPLE:<br>0.2 ± 0.1 k/mg Hb<br>OPLP:<br>0.4 ± 0.4 k/mg Hb<br><br>Week 8:<br>N:<br>0.7 ± 0.1 k/mg Hb<br>C:	Antioxidant activity

	0.9 ± 0.1 k/mg Hb<br>OPLE:<br>0.6 ± 0.1 k/mg Hb<br>OPLP:<br>0.9 ± 0.3 k/mg Hb<br><br>Week 16:<br>N:<br>0.6 ± 0.2 k/mg Hb<br>C:<br>1.1 ± 0.4 k/mg Hb<br>OPLE:<br>0.9 ± 0.4 k/mg Hb<br>OPLP:<br>0.7 ± 0.2 k/mg Hb
Glutathione peroxidase (GPx) activity	Week 0:<br>N:<br>8.9 ± 0.8 k/mL<br>C:<br>9.3 ± 1.4 k/mL<br>OPLE:<br>9.5 ± 2.4 k/mL<br>OPLP:<br>9.1 ± 2.2 k/mL<br><br>Week 8:<br>N:<br>5.1 ± 1.8 k/mL<br>C:<br>6.4 ± 2.0 k/mL<br>OPLE:<br>5.8 ± 1.8 k/mL<br>OPLP:<br>7.2 ± 1.4 k/mL

		Week 16:<br>N:<br>3.1 ± 1.5 k/mL<br>C:<br>2.5 ± 1.8 k/mL<br>OPLE:<br>2.7 ± 1.0 k/mL<br>OPLP:<br>3.2 ± 1.6 k/mL
	MDA level	Week 0:<br>N:<br>6.5 ± 0.9 nmol/mL<br>C:<br>5.8 ± 3.3 nmol/mL<br>OPLE:<br>6.8 ± 2.2 nmol/mL<br>OPLP:<br>5.5 ± 1.4 nmol/mL<br><br>Week 8:<br>N:<br>2.5 ± 1.0 nmol/mL<br>C:<br>1.8 ± 0.2 nmol/mL<br>OPLE:<br>1.8 ± 0.5 nmol/mL<br>OPLP:<br>1.7 ± 0.4 nmol/mL<br><br>Week 16:<br>N:<br>2.7 ± 1.2 nmol/mL<br>C:<br>2.4 ± 0.4 nmol/mL<br>OPLE:<br>2.2 ± 0.4 nmol/mL<br>OPLP:

				2.5 ± 0.6 nmol/mL	
			MDA in liver homogenate	Week 16:<br>N:<br>1.6 ± 0.5 nmol/g ww<br>C:<br>3.2 ± 0.9 nmol/g ww<br>OPLE:<br>2.2 ± 0.5 nmol/g ww<br>OPLP:<br>2.4 ± 0.8 nmol/g ww	
[10]	Female Sprague – Dawley rats	Water (Control)<br><br>OPLE:<br>-Ethanolic extract	Subacute oral toxicity study	Feeding for 28 days with 0.5, 1, and 2 g/kg/day of OPLE:<br><br>No changes in behaviour, respiration, neuronal responses<br><br>No effects on liver functions, renal functions, metabolic and hematologic status.<br><br>Slight increase in the relative weight of the liver. Relative weight of other organs not affected	Toxicity effect
			Acute oral toxicity study	Safe with LD50 < 5 g/kg.	
[11]	Sprague Dawley strain albino rats	OPLE:<br>10% (w/w) formulated crude extract<br>(5 g of the methanol oil palm leaf extract in 50 g of yellow soft paraffin)	Microbial count analysis	Day 16<br>Treated rats: 102 CFU.<br>Control rats: 104 CFU.	Antimicrobial activity
			In vivo wound healing activity	Faster rate of wound closure observed in the OPLE treated group.<br><br>Percentage of wound closure:<br><br>Day 4:	Wound healing activity

		Control:<br>Yellow soft paraffin		Control group: 15%<br>OPLE Treated group: 20%<br><br>Day 8:<br>Control group: 30%<br>OPLE Treated group: 40%<br><br>Day 12:<br>Control group: 50%<br>OPLE Treated group: 65%<br><br>Day 16:<br>Control group: 70%<br>OPLE Treated group: 95%<br><br>Complete wound closure:<br>Treated group: 16 days.<br>Control group: 25 days.	
			Histological analysis/ Histopathology Analysis: Microbial examination of granulated skin tissue	Complete loss of superficial epithelium and inflammatory exudates were observed in both groups. The clumps of yeast were higher in the control group compared to the treated rats.	Fungicidal activity
[8]	Male – Wistar Kyoto rats	Positive control:<br>Acetylcholine<br><br>Methanolic extracts:<br>Palm frond<br>Papaya<br>Chili<br>Lemongrass	Vascular function studies	Vascular relaxing activity of various plant extracts in endothelium-intact aortic rings:<br><br>OPLE: 84%<br>Papaya shoot: 45%<br>Green chili: 35%<br>Lemongrass: 32%<br>Acetylcholine: 80%	Vascular relaxation

Vascular relaxing activity of various plant extract in per-fused mesenteric vascular bed.:

OPLE: 70%
Papaya shoot: 35%
Green chili: 22%
Lemongrass: 55%
Acetylcholine: 60%

Loss of relaxing activity of plant extracts following de-endothelialisation:

OPLE: 109 ± 1%
Papaya shoot: 96 ± 0.4%
Green chili: 83 ± 1 %
Lemongrass: 82 ± 2 %

Loss of relaxing activity of plant extracts following inhibition of NO using NOLA
In endothelium-intact aortic rings:

OPLE: 100 ± 0.1%
Papaya shoot: 107 ± 1%
Green chili: 58 ± 1%
Lemongrass: 87 ± 1%

Loss of relaxation of various plant extracts following inhibition of NO with NOLA and cyclooxygenase with indomethacin in the perfused mesenteric vascular bed:

OPLE: 80 ± 4*%
Papaya shoot: 56 ± 6%

				Green chili: 26 ± 11%<br>Lemongrass: 34 ± 5%	
[15]	Male – Wistar Kyoto rats	WKY:<br>+Control<br>+OPLE<br>+Captopril<br>+L-NAME<br>+L-NAME+OPLE<br>+L-NAME+captopril	Plasma MDA concentration	Week 0 (μmol/L):<br><br>+Control: 5.85±0.18<br>+OPLE: 5.30±0.32<br>+Captopril: 5.67±0.99<br>+L-NAME: 4.66±0.86<br>+L-NAME+OPLE: 6.57±0.43<br>+L-NAME+captopril: 5.44±0.28<br><br>Week 6 (μmol/L):<br><br>+Control: 7.06±0.09<br>+OPLE: 5.62±0.61<br>+Captopril: 6.36±0.03<br>+L-NAME: 8.35±0.49<br>+L-NAME+OPLE: 6.59±0.36<br>+L-NAME+captopril: 6.8±0.38<br><br>Week 12 (μmol/L):<br><br>+Control: 78.74±0.67<br>+OPLE:	Antioxidant activity

		7.50±0.51<br>+Captopril:<br>7.83±0.49<br>+L-NAME:<br>8.43±0.84<br>+L-NAME+OPLE:<br>7.17±0.85<br>+L-NAME+captopril:<br>8.29±0.73	
	SOD activity in erythrocytes	Week 0 (mU/mL):<br><br>+Control:<br>13.0±0.8<br>+OPLE:<br>14.6±1.2<br>+Captopril:<br>14.0±1.0<br>+L-NAME:<br>13.8±0.9<br>+L-NAME+OPLE:<br>12.8±1.4<br>+L-NAME+captopril:<br>13.3±1.0<br><br>Week 6 (mU/mL):<br><br>+Control:<br>22.8±1.1<br>+OPLE:<br>22.7±1.0<br>+Captopril:<br>21.8±0.6<br>+L-NAME:<br>12.0±0.9<br>+L-NAME+OPLE:<br>9.9±0.7<br>+L-NAME+captopril:	

		12.6±1.1<br><br>Week 9 (mU/mL):<br><br>+Control:<br>16.2±1.4<br>+OPLE:<br>18.0±0.7<br>+Captopril:<br>14.8±1.9<br>+L-NAME:<br>8.1±0.4<br>+L-NAME+OPLE:<br>6.5±0.9<br>+L-NAME+captopril:<br>7.2±0.8<br><br>Week 12 (mU/mL):<br><br>+Control:<br>20.1±0.7<br>+OPLE:<br>19.5±1.0<br>+Captopril:<br>18.6±0.6<br>+L-NAME:<br>10.3±1.2<br>+L-NAME+OPLE:<br>10.7±0.8<br>+L-NAME+captopril:<br>10.8±0.8
	Catalase activity in erythrocytes	Week 0 (mU/mL):<br><br>+Control:<br>11.4±0.9

+OPLE:<br>13.2±0.7<br>+Captopril:<br>13.5±0.6<br>+L-NAME:<br>11.8±0.7<br>+L-NAME+OPLE:<br>12.4±0.4<br>+L-NAME+captopril:<br>12.9±0.5<br><br>Week 6 (mU/mL):<br><br>+Control:<br>13.6±1.1<br>+OPLE:<br>13.6±1.3<br>+Captopril:<br>13.4±0.3<br>+L-NAME:<br>15.0±0.7<br>+L-NAME+OPLE:<br>12.9±0.6<br>+L-NAME+captopril:<br>12.5±0.5<br><br>Week 9 (mU/mL):<br><br>+Control:<br>12.8±0.4<br>+OPLE:<br>15.6±0.7<br>+Captopril:<br>14.0±0.5<br>+L-NAME:<br>11.5±0.6<br>+L-NAME+OPLE:

	12.6±0.7<br>+L-NAME+captopril:<br>13.1±0.6<br><br>Week 12 (mU/mL):<br><br>+Control:<br>13.9±0.5<br>+OPLE:<br>17.6±0.8<br>+Captopril:<br>13.1±0.8<br>+L-NAME:<br>13.8±0.5<br>+L-NAME+OPLE:<br>15.4±0.9<br>+L-NAME+captopril:<br>14.3±0.3
GPx in erythrocytes	Week 0 (mU/mL):<br><br>+Control:<br>19.8±3.8<br>+OPLE:<br>20.7±3.2<br>+Captopril:<br>16.5±0.9<br>+L-NAME:<br>22.6±1.8<br>+L-NAME+OPLE:<br>21.4±5.7<br>+L-NAME+captopril:<br>22.1±3.1<br><br>Week 6 (mU/mL):

+Control:<br>18.8±2.5<br>+OPLE:<br>25.2±3.8<br>+Captopril:<br>21.1±5.1<br>+L-NAME:<br>10.0±1.3<br>+L-NAME+OPLE:<br>14.0±4.0<br>+L-NAME+captopril:<br>14.2±2.0<br><br>Week 9 (mU/mL):<br><br>+Control:<br>20.0±5.8<br>+OPLE:<br>22.6±4.9<br>+Captopril:<br>14.1±1.8<br>+L-NAME:<br>15.9±1.8<br>+L-NAME+OPLE:<br>16.5±1.7<br>+L-NAME+captopril:<br>16.6±2.7<br><br>Week 12 (mU/mL):<br><br>+Control:<br>24.3±4.2<br>+OPLE:<br>27.0±3.2<br>+Captopril:

	26.2±4.4<br>+L-NAME:<br>31.2±3.2<br>+L-NAME+OPLE:<br>18.9±1.9<br>+L-NAME+captopril:<br>17.2±0.9
Serum nitric oxide level	Week 0 (ppm):<br><br>+Control:<br>0.414±0.024<br>+OPLE:<br>0.347±0.014<br>+Captopril:<br>0.381±0.023<br>+L-NAME:<br>0.339±0.034<br>+L-NAME+OPLE:<br>0.319±0.044<br>+L-NAME+captopril:<br>0.402±0.008<br><br>Week 6 (ppm):<br><br>+Control:<br>0.548±0.009<br>+OPLE:<br>0.409±0.109<br>+Captopril:<br>0.573±0.024<br>+L-NAME:<br>0.104±0.056<br>+L-NAME+OPLE:<br>0.197±0.053<br>+L-NAME+captopril:<br>0.585±0.082

		Week 9 (ppm):<br><br>+Control:<br>0.417±0.053<br>+OPLE:<br>0.468±0.096<br>+Captopril:<br>0.455±0.060<br>+L-NAME:<br>0.276±0.061<br>+L-NAME+OPLE:<br>0.283±0.084<br>+L-NAME+captopril:<br>0.742±0.123<br><br>Week 12 (ppm):<br><br>+Control:<br>0.384±0.038<br>+OPLE:<br>0.362±0.002<br>+Captopril:<br>0.525±0.071<br>+L-NAME:<br>0.210±0.074<br>+L-NAME+OPLE:<br>0.531±0.086<br>+L-NAME+captopril:<br>0.44±0.072	
	MDA level in hearts	(nmol/g of tissue)<br>+Control:<br>23.49±2.17<br>+OPLE:<br>18.77±1.99<br>+Captopril:	Antihypertensive and cardiovascular effects

	17.54±0.41<br>+L-NAME:<br>18.73±3.17<br>+L-NAME+OPLE:<br>13.01±1.20<br>+L-NAME+captopril:<br>18.31±2.05
SOD level in hearts	(units/mg of protein)<br>+Control:<br>3.91±0.07<br>+OPLE:<br>3.69±0.011<br>+Captopril:<br>3.91±0.004<br>+L-NAME:<br>4.54±0.80<br>+L-NAME+OPLE:<br>3.25±0.21<br>+L-NAME+captopril:<br>3.64±0.36
Catalase level in hearts	(k/mg of protein)<br>+Control:<br>4.39±0.56<br>+OPLE:<br>6.08±1.02<br>+Captopril:<br>5.42±1.02<br>+L-NAME:<br>5.02±0.99<br>+L-NAME+OPLE:<br>4.11±0.53<br>+L-NAME+captopril:<br>4.31±0.78
GPx level in hearts	(units/mg of protein)<br>+Control:<br>3.35±0.71<br>+OPLE:

		4.16±0.71<br>+Captopril:<br>5.20±1.14<br>+L-NAME:<br>5.33±0.48<br>+L-NAME+OPLE:<br>4.69±0.44<br>+L-NAME+captopril:<br>4.84±0.36
	Systolic Blood pressure	Co-administration of L-NAME with OPLE or captopril significantly attenuated BP increase.<br><br>OPLE showed no hypotensive effects in normotensive rats.
	Final body weight and heart weight of rats	L-NAME groups showed significantly lower weight gains by about 30% compared to normal groups.<br><br>Captopril treatment on normal and L-NAME rats further reduced weight gains.
	Changes in coronary small arteries	Significantly thickened and changes in the wall to lumen ratio compared to control rats after 12 weeks of NO deficiency. There were decreases in artery wall thickness with co-administration of OPLE or captopril resulting in decreased wall to lumen ratios to normal values.<br>Neither OPLE nor captopril affected aorta wall thickness and coronary artery wall to lumen ratio in all normotensive rats.

			Percentage of myocardial fibrosis and myocardial fibre thickness	NO deficiency dramatically increased the fibrosis to 60% compared with control rats.<br><br>Only L-NAME+captopril group demonstrated near-normal myocardium with no increase in thickness.	
[16]	Sprague Dawley albino rats	-Treatment group (5g of 10% OPLE in 50g of yellow soft paraffin)<br>-Control group	In vivo wound healing activity	Significant difference in wound closure observed in treated group from day 4 onwards, also much faster wound closure rate compared with control.<br><br>Complete wound closure<br>Treatment group: 16 days.<br>Control group: 25 days.	Wound healing activity
			Histological analysis	Treatment group: significant increase in collage deposition with fewer macrophages and more fibroblasts.<br><br>Control group: decreased collagen content.	
			Gelatin Zymography	Treatment group: expression of MMPs were high followed by reduction in later days of healing.<br><br>Control group: expression of MMPs was more in control rats compared to treatment rats and persists until day 12.<br><br>Major MMPs observed are MMP 8 and MMP 9.	

References

1. Hui, A.C., C.S. Foon, and C.C. Hock, Antioxidant activities of Elaeis guineensis leaves. J. Oil Palm Res., 2017. 29(Sept.): p. 343-351.
2. Soundararajan, V. and S. Sreenivasan, Antioxidant Activity of Elaeis guineensis Leaf Extract: An Alternative Nutraceutical Approach in Impeding Aging. APCBEE Proc., 2012. 2(3rd International Conference on Biotechnology and Food Science, 2012): p. 153-159.
3. Han, N.M. and C.Y. May, Determination of antioxidants in oil palm leaves (Elaeis guineensis). Am. J. Appl. Sci., 2010. 7(9): p. 1243-1247.
4. Ahmad, N., et al., Determination of total phenol, flavonoid, antioxidant activity of oil palm leaves extracts and their application in transparent soap. J. Oil Palm Res., 2018. 30(June): p. 315-325.
5. Che, Z.M.S., et al., In Vitro Wound Healing Potential of Flavonoid C-Glycosides from Oil Palm (Elaeis guineensis Jacq.) Leaves on 3T3 Fibroblast Cells. Antioxidants (Basel), 2020. 9(4).
6. Salleh, M.N., et al., Inhibition of Low-Density Lipoprotein Oxidation and Up-Regulation of Low-Density Lipoprotein Receptor in HepG2 Cells by Tropical Plant Extracts. J. Agric. Food Chem., 2002. 50(13): p. 3693-3697.
7. Che, Z.M.S., et al., Metabolite Characterization and Correlations with Antioxidant and Wound Healing Properties of Oil Palm (Elaeis guineensis Jacq.) Leaflets via (1)H-NMR-Based Metabolomics Approach. Molecules, 2020. 25(23).
8. Abeywardena, M., et al., Polyphenol-enriched extract of oil palm fronds (Elaeis guineensis) promotes vascular relaxation via endothelium-dependent mechanisms. Asia Pac. J. Clin. Nutr., 2002. 11(Suppl.): p. S467-S472.
9. Irine, R., et al., Antioxidant and hypocholesterolemic effects of Elaeis guineensis frond extract on hypercholesterolemic rabbits. ASEAN Food J., 2003. 12(3): p. 137-147.
10. Ibraheem, Z., et al., Toxicity, phytochemical content and antioxidant activity assessment studies for a standardized ethanolic fraction of palm oil leaf extract. Pharmacognosy Communications, 2012. 2: p. 21-30.
11. Sreenivasan, S., et al., Wound healing potential of Elaeis guineensis Jacq leaves in an infected albino rat model. Molecules, 2010. 15: p. 3186-3199.
12. Bakhsh, A., N.M. Mustapha, and S. Mohamed, Catechin-rich oil palm leaf extract enhances bone calcium content of estrogen-deficient rats. Nutrition, 2013. 29(4): p. 667-672.
13. Mohamed, S., T. Lee Ming, and J.M. Jaffri, Cognitive enhancement and neuroprotection by catechin-rich oil palm leaf extract supplement. Journal of the Science of Food and Agriculture, 2013. 93(4): p. 819-827.
14. Rosalina Tan, R.T., et al., Polyphenol rich oil palm leaves extract reduce hyperglycaemia and lipid oxidation in STZ-rats. Int. Food Res. J., 2011. 18(1): p. 179-187.
15. Jaffri, J.M., et al., Antihypertensive and cardiovascular effects of catechin-rich oil palm (Elaeis guineensis) leaf extract in nitric oxide-deficient rats. J Med Food, 2011. 14(7-8): p. 775-83.
16. Sasidharan, S., S. Logeswaran, and L.Y. Latha, Wound healing activity of Elaeis guineensis leaf extract ointment. International journal of molecular sciences, 2012. 13(1): p. 336-347.
